# Older research participants are motivated to receive genetic results for the benefit of younger relatives

**DOI:** 10.1038/s41431-025-01940-8

**Published:** 2025-09-22

**Authors:** Amanda M. Willis, Florence Chiew, Philomena Horsley, Sommon Klumsathian, Ezekiel Ling, Chris Jacobs, Paul Lacaze, Jane Tiller, Mary-Anne Young

**Affiliations:** 1 Clinical Translational and Engagement Platform, Garvan Institute of Medical Research, Darlinghurst, NSW Australia; 2 School of Clinical Medicine, UNSW Medicine & Health, St Vincent’s Healthcare Clinical Campus, Faculty of Medicine and Health, UNSW Sydney, Sydney, NSW Australia; 3 Melbourne School of Population and Global Health, The University of Melbourne, Melbourne, Australia; 4 Graduate School of Health, University of Technology Sydney, Sydney, NSW Australia; 5 School of Health Sciences, University of Surrey, Guildford, UK; 6 School of Public Health and Preventive Medicine, Monash University, Melbourne, VIC Australia

**Keywords:** Genetics research, Ethics, Genetic testing

## Abstract

The benefit of returning medically actionable genetic research findings is widely recognised. However, there is uncertainty regarding the return of results to older people (>70 years), given the reduced actionability in this age group. The goal of this study was to assess whether older people were motivated to receive genetic results, primarily for the benefit of younger relatives. Semi-structured interviews were conducted with individuals aged ≥70 enrolled in the ASPirin in Reducing Events in the Elderly (ASPREE) study. Participants received medically actionable genetic results in Hereditary Breast and Ovarian Cancer, Lynch syndrome or Familial Hypercholesterolaemia genes. Data were analysed using reflexive thematic analysis. Sixteen individuals were interviewed (mean age 82 years). While participants recognised the limited actionability of the genetic results for themselves, the perceived benefits for younger family members motivated them to receive genetic findings and share with relatives. Participants reported positive experiences of receiving genetic results, underpinned by their existing relationship with the ASPREE trial, and reported that their age promoted their adaptation to results. These findings illustrate positive impact from returning genetic research results to older research participants and suggest that older people desire genetic information to benefit their younger family members.

## Introduction

Advances in the use of genomic technologies in research mean increasing numbers of research participants are being identified with medically actionable genetic research results as secondary findings (SFs). There is a growing ethical imperative for researchers to offer this information to participants, given the potential health benefits for participants and their relatives [1]. Further, participants report a preference to be informed of their results for reasons of personal and clinical utility [2, 3]. Evidence also indicates that most individuals have positive experiences of receiving research results and express high intention to disseminate genetic information to family members [4].

However, it has been suggested that the age of research participants may impact whether and how genomic research results are returned [5–7]. Among older individuals, there is less certainty regarding the clinical utility and actionability of genetic information, particularly in the setting of opportunistic screening for SFs. However, the extended impact and potential benefits of this information for younger family members are substantial [6]. Additional challenges associated with returning results to older participants include the potential impact of cognitive decline and other morbidity, and the possibility that participants may be deceased by the time results are returned [5]. These issues raise questions regarding the ethical imperative and practicality of offering SFs to older people, if primarily for the benefit of their younger family members.

Given historical exclusion of older people from research [8, 9], there is limited literature reporting the views and experiences of older people participating in genetic research, and the impact of returning SFs in this age group. The views of older research participants may help guide how research practice progresses in this area, including consideration of the potential benefits of returning genetic information to older people and their relatives, and the potential challenges when returning genetic information in this setting.

The ASPirin in Reducing Events in the Elderly (ASPREE) trial is an international clinical trial of daily low-dose aspirin for primary prevention in individuals over the age of 70, with a genetic sub-study to determine the rate of medically actionable variants in a healthy older population [10, 11]. At an average age of 82 years, ASPREE participants may have lived through much of the risk associated with SFs identified by opportunistic screening. The decision was taken to return these SFs to ASPREE participants, largely for the potential health benefits to other family members. This study aimed to explore the views and experiences of these older research participants who received SFs from the ASPREE trial.

## Materials and methods

A qualitative approach was taken, given the limited literature regarding older people’s experiences of research SFs and the exploratory nature of the research [12]. Ethics approval for the return of genetic results in certain genes (*BRCA1*, *BRCA2*, *PALB2*, *MLH1*, *MSH2*, *MSH6*, *PMS2*, *LDLR*, *PCSK9*, *APOB*) and associated psychosocial research was granted by Alfred Hospital Human Research Ethics Committee (390/15).

### Setting and participants

Participants recruited to the ASPREE Healthy Ageing Biobank [13] provided consent for genetic research and recontact if medically actionable genetic findings were identified (Box 1).

**Fig. 1:**
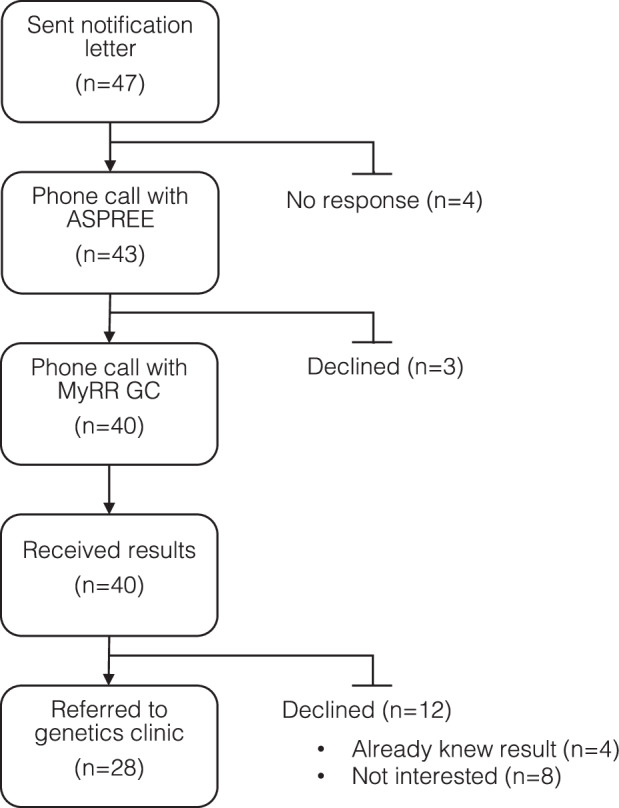
ASPREE results return pathway showing participant numbers at each stage. GC genetic counsellor.

Targeted genome sequencing was performed on Australian ASPREE participants and SFs were returned in collaboration with the national genetic counselling service for research participants, My Research Results (MyRR) [14]. ASPREE participants with SFs identified were notified of the availability of results by letter, which invited them to contact the ASPREE study team. The ASPREE study team checked interest in receiving SFs and then scheduled a telephone appointment with a genetic counsellor from MyRR for disclosure of results for participants who elected to continue (Fig. 1). MyRR genetic counsellors facilitated referral to a clinical genetics service for diagnostic confirmation of research results, personalised risk assessment and cascade testing.

Box 1 Information provided to ASPREE Biobank participants regarding SFs If our research uncovers any significant information specific to your health, our ASPREE Biospecimen Governance Committee may decide to authorise someone to contact you and offer you access to this information. You may decline the information. If you wish to be given this information a qualified person will explain it to you. You should also consider whether this information should be made known to your family members. Sharing these findings could help avoid similar medical problems in your family.

### Recruitment

Individuals were eligible to participate in this qualitative study if they: (i) were still enroled in ASPREE (some participants withdrew over time); and (ii) had received genetic results via the pathway described above. Individuals were excluded if they had declined to proceed with the results return pathway after disclosure.

A purposive sampling approach was taken with the aim to ensure participants were varied with regard to sex and SF received. Participants were sent information about the qualitative study and a letter inviting them to contact the research team if they wished to participate in an interview. If participants did not respond to the letter after 4 weeks, two attempts were made to contact them by phone. Participants who expressed interest in the study were contacted by phone to schedule an interview.

### Data collection

Individual semi-structured interviews were conducted by phone by an experienced qualitative researcher not involved in the return of results: PH (medical anthropologist) or FC (sociologist and psychologist). An interview guide was developed that focused on participants’ experience of receiving genetic information from ASPREE, including the results return process, perceived value of genetic information, family communication and impact (see Supplementary Materials). Verbal consent to participate was recorded at commencement of the interview. Interviews were audio recorded and transcribed verbatim by a professional transcription service (https://www.rev.com/). All identifying information was removed from the interview transcripts and pseudonyms assigned.

### Data analysis

An inductive, reflexive thematic analysis was undertaken. Data analysis occurred concurrent with data collection to allow for flexibility in applying the interview guide to address new concepts relevant to the research question [15]. No changes were made to the interview guide during data collection. However, the analysis team met and discussed the first three transcripts and agreed to explore the role of age and the familial impact of results in more depth in subsequent interviews based on participant accounts.

Each interview was coded by at least two members of the study team (SK/EL: Master of Genetic Counselling students; AW/MAY genetic counsellors; FC: sociologist and psychologist). A collaborative coding approach was used to support student researchers, as well as incorporate multiple perspectives to produce a richer and more nuanced interpretation of the data [16]. All coders first read the transcripts to familiarise themselves with the data, then read and applied initial codes to the transcripts. The study team met regularly to discuss coding and iteratively organised codes into categories, concepts and themes that represented important dimensions of participants’ experiences [15, 17].

Recruitment continued until the research team agreed that sufficient data had been collected to achieve a comprehensive understanding of the research topic. The study team, particularly those with genetic counsellor training, acknowledged that their clinical experience and attitudes to genetic information may influence the analysis and interpretation of data. The inclusion of study team members from a non-genetics background and collaborative coding approach enhanced interpretation of the data. Quotes are presented using pseudonyms, participant age at result disclosure and sex.

## Results

Twenty-five ASPREE participants were invited to participate in the interview study, and 16 completed interviews, with an average interview length of 32 min (range 9–68 min). Participant characteristics are summarised in Table 1. Nine of the 16 participants were female, the majority of participants received a result indicating risk for hereditary breast/ovarian cancer and the majority had children. Two participants, each with a *BRCA1* variant, were already aware of their genetic result from previous clinical testing.

**Table 1:** Interview participant characteristics (*n* = 16).

	*N*
Average age (range)	82 (78–89)
Sex	
Female	9
Male	7
Children	
Yes	15
No	1
Result	
BRCA1	5
BRCA2	6
PALB2	2
MSH6	2
LDLR	1
Result previously known	
No	14
Yes	2

Three main themes were identified by the analysis: (i) the influence of life stage and shifting perspectives on participant experiences of receiving research results (ii); participants’ feeling a duty of care for their family; and (iii) how the reciprocal research relationship fostered by ASPREE impacted participant experiences of receiving research results.

### Life stage and shifting perspectives

Participants’ age and life experience influenced their approach to research and receiving genetic information, with participants largely taking a pragmatic view of the research genetic testing.

*Oh, I figured they’d do genetic testing down the line somewhere. I didn’t quite know whether I’d still be here when they did it. But I knew that it would be done. It’s like buying a raffle ticket, love. If they find something, they find something. If they don’t find something, well, we live with it*.

Judy, 82, female

Participants understood the limited health impact of the genetic information for themselves. There was little change to their medical care as most had lived through the bulk of their cancer risk, had previously been diagnosed with cancer, or were already appropriately managed.

*I don’t think I have felt any anxiety for myself over this at all because, well, you know, I’m too old now, it’s not gonna matter*.

Martha, 89, female

*It didn’t worry me in that respect, but it alerted me to something that I didn’t know I’d had. But from what I’ve got of the reading of it, it wasn’t over urgent because I’d had all my ovaries removed and everything like that*.

Elaine, 81, female

However, participants still emphasised the importance of maintaining good health and early intervention in older age.

*You know, your sort of lifestyle, amount of stress in your life, and all those things that tend to be just as important in whether you end up with some of these complaints or not*.

Anthony, 79, male

*The main thing, I think, is that you have to stay healthy, like anybody should. If the cancer is going to come, it’s sneaky, it just will appear. But as long as you get it early, you have a very good chance of survival these days*.

Margarita, 78, female

Participants described minimal emotional impact from receiving their results, for example one participant stated, ‘it didn’t worry me one bit’. Age and life experience were described as a source of resilience, which enhanced one’s capability to deal with life’s challenges.

*As you get older, you don’t worry about things either. You become more patient and more relaxed and more knowledgeable because of experience in your life. And it makes things easier for you. It makes you stronger, all the experiences of life*.

Anika, 82, female

Participants also discussed how, with age and a life well lived, comes an acceptance of death that made the genetic information less concerning.

*It’s told me I have a mild chance of getting some sort of cancer. I’m not worried about that because at my age I don’t give a booger. Not that I’m wanting to go out, but I’ve had a good life*.

Dylan, 81, male

While acknowledging the limited impact of the information for themselves, given their age, participants recognised that the information had substantial impact for younger family members.

*When you get to my age, you think “Is it important to me at my time of life?” No, it doesn’t matter to me, but it does for the kids*.

Bea, 86, female

### Duty of care for family

All participants spoke about the value and importance of the information for their family and had shared results with at least one family member. Many participants stated their family was the main reason for taking up the offer of genetic results and undergoing diagnostic confirmatory testing.

*I only wanted to know for their [family’s] sake, just to pass that information onto them. If they need it, it’s there. Just a helpful warning for them*.

Pamela, 80, female

Many also described a sense of responsibility to ensure that family members were informed of the genetic information and given the ‘opportunity’ to have their own testing and take action to manage their health.

*I immediately wrote to them [brothers] and I told them that they must tell their children and their children must tell their children and so on so that every member of the family, male or female, knows about it*.

Martha, 89, female

*When I get the paperwork, I’ll just send it on to them, so that anyone that wants to can go and get a test to see if they’ve got this gene that is defective*.

Richard, 85, male

Sometimes, participants felt it was not their place to inform certain family members, particularly grandchildren, nieces and nephews, and relied on others to pass on information.

*I’ve told all my kids, and I would hope that they would pass that on to my grandkids. I thought it was probably their duty to tell the grandkids, rather than me waiting to tell the grandkids when perhaps their parents didn’t want them to know something*.

Anthony, 79, male

*I told my son what it was, and I said it mainly affects girls. He said if it comes to it, I’ll explain to [daughter], but I don’t think I need to tell her anything now. I had to leave it with him*.

Elaine, 81, female

While participants felt responsible for informing family members and wanted to see the information used, many discussed that they could not force family members to undergo testing or take action.

*I left it up to them [children] to do what they wanted to do. It’s their lives, not mine to interfere in*.

Vincent, 83, male

*I probably would’ve preferred if she [daughter] got tested, but she’s 44. She’s got to make her own decisions*.

Brittany, 79, female

For the most part, participants saw the information as beneficial for their family and did not report negative outcomes from sharing the information with family members.

*It’s actually very, very positive. Very useful. It has definitely saved them a lot of trouble and hassle. For example, my daughter and her daughters. If they go down the testing track it and prevent it possibly with early detections. There’s a number of benefits one can construct with your imagination*.

Nicholas, 82, male

While still seeing the information as positive for the family, a couple of male participants with daughters did express feelings of guilt at having passed on a genetic variant that had a greater impact for daughters than for themselves.

*My girls are getting warned, I think it’s all good… You know, it’s just one of those things when you feel a bit guilty that, “Hey, it came from me”.*

Lou, 81, male

### Reciprocal research relationship

Participants all reported very positive experiences of the ASPREE trial. They described a reciprocal research experience, including routine feedback of results from tests done as part of the trial. This foundation of reciprocity and trust facilitated the return of genetic information and meant that genetic information was well received by participants.

*I thought it would be just, you know, a bit of a one-way thing. You sort of give this information, all these faceless people pile it all up, and stick it through the computer and it all flies out the end as a sausage… But no, I’m very thankful for the care and concern you’ve [ASPREE team] taken*.

Anthony, 79, male

*ASPREE was a very well thought out project where you got information back from time to time, and I would’ve expected that if anything had been found, such as it was, that it would be reported back to me*.

Richard, 85, male

This was despite the unexpected nature of the genetic results, given their history of good health and variable recollection of consent to research genetic testing.

*I just thought at the time it was only what the difference was between taking aspirin and not taking aspirin. That was all I thought in the first place that it was going to be about. I didn’t know it was going to delve into my history, but it didn’t worry me*.

Elaine, 81, female

*I certainly didn’t expect them to find I carry the cancer thing because I don’t come from a family where cancer is a big problem*.

Bea, 86, female

Participants also described a sense of satisfaction and meaning from contributing to research.

*Well obviously the trial produced the result, so my parting piece will probably be the same as a piece of sand on Bondi Beach, but without all of those pieces of sand it wouldn’t be a Bondi Beach, would it?*

Nicholas, 82, male

Participants reflected positively on the process used to return genetic results, describing it as ‘gentle’ and ‘respectful’, with clear communication and adequate support.

*I have been very impressed the way you’ve handled it. The emails, the letters that sometimes I will receive as a follow-up, like a hard copy. Everything. I think it’s been really good*.

Lou, 81, male

*There’s nothing I can think I could criticise or find fault with them and the process at all*.

Christine, 84, female

For many participants, the return of genetic results added value to their participation, and a number expressed gratitude for the information.

*Getting this information now just shows how worthwhile it has been to be in the trial, because I’d be none-the-wiser otherwise*.

Richard, 85, male

*I’m thankful to have been part of the program. If they wanna give me more information that’s genetically based, that’s fine. I’m glad that they’re using the material that they’ve got to the full benefit*.

Shane, 79, male

## Discussion

This study explored the experiences of older research participants who participated in a clinical trial and received SFs from research genetic testing. Other studies returning SFs to participants have included participants of varied ages, although the average age of participants is younger than the ASPREE group, typically around 40–60 [18–22]. Age-related differences are also rarely explored, so data regarding the views of older participants regarding SFs is lacking. Thus, the results of our study provide insight into the views of older research participants on genetic information, family and responsibility. Our findings suggest that age and life experience can be a source of resilience when receiving research results. Participants emphasised the importance of the information for their younger family members, and the personal value of genetic research results.

The value of receiving SFs reported by participants in this study challenges assumptions that older participants are less likely to benefit from health information or intervention and more likely to experience harms (e.g. distress, over screening) because of their age [5–7]. Differential treatment or exclusion of older people occurs in many health and research contexts and has been demonstrated to have a negative impact on health outcomes [23, 24]. Negative beliefs about ageing alone have also been demonstrated to impact health outcomes [23]. One study returning research SFs found no differences in preferences or consent rates for SFs by age [21]. Other studies reported that older age has been associated with increased interest in genetic information [7, 25, 26]. This highlights the importance of considering assumptions and structural factors influencing participation and treatment of older people in genetic health and research settings.

Genetic testing is most commonly provided in settings requiring clear medical utility to justify use of often limited funding and resources. This raises whether SFs should be returned to older people, primarily benefit of younger family members. Similar issues have been addressed in the palliative care setting, where genetic testing or DNA banking is performed largely for the benefit of family members. Current research suggests access to risk information for relatives can be a driver for genetic testing and of significant value to palliative patients [27, 28], consistent with our findings. Despite concerns about causing distress by raising genetics in the palliative setting [29], there is support for family-centred integration of genetics into palliative care, given the significant implications of missing an opportunity to identify familial risk [30, 31]. The return of SFs to older research participants using existing genomic datasets could represent another opportunity to identify families at high risk, in an arguably less resource-intensive and lower-stakes setting than palliative care.

All participants in our study shared their results with at least one family member and reported family as a key motivator for receiving results, consistent with research demonstrating that value for relatives is a common driver for genetic testing [26, 32]. High rates of sharing research results have been reported in other contexts [33] and is important for facilitating cascade testing in relatives. While family communication about SFs is common [4], one study found participants over 45 years old were less likely to tell *all* relatives about their results compared to those under 45 years [20]. This could speak to the patterns of sharing information mentioned by participants in this study, who felt their children should inform grandchildren.

Older research participants in this study understood the limited clinical utility of the genetic results for themselves and described their age and life experience as facilitating their adaptation to the information. Previous research raised concerns that providing genetic risk information could perpetuate misconceptions about the clinical benefit of screening for older people, leading to unnecessary tests or worry if screening is not offered [7]. However, this was not observed in our study and other research has reported underutilisation of health care more likely than overutilisation in response to SFs from research [18]. The knowledge that the health and other (e.g. insurance, reproductive) impacts of the information is minimal may make the information easier to cope with [7, 34], as described by participants in this study. It could also be argued that older people have built resilience and capacity to adapt to genetic information.

This model of returning results, which enabled people to choose whether to receive genetic information, was well received by interviewees. Being able to choose what information to receive from genetic testing has been identified as important to individuals across many contexts and age groups [35]. ASPREE participants had also built a long-term relationship with the research team, which facilitated ongoing trust and mutual goodwill. In this context, participants described the return of genetic information as a positive experience that enhanced their research participation. Previous studies have discussed the value of research participation and volunteering on well-being, particularly in older age [7, 36, 37]. Participants also reported value from receiving their personal genetic information for its own sake, as well as other aspects of personal utility, as observed in other studies [2, 19, 32]. These findings suggest that this model of participant engagement and return of results could be used as a model for other studies.

Despite the benefits described by participants in this study, returning genetic research results to an ageing cohort has practical challenges. Results were provided by genetic counsellors at no cost to participants, with follow-up care for self and family members provided at no cost in the Australian public health system. Responses to the provision of genetic information may be different in other contexts, although literature suggests participant experiences of receiving SFs are largely positive [4]. Participants potentially being unable to receive SFs because they are deceased or experiencing significant cognitive decline also presents challenges. Other studies have successfully disclosed unexpected results to a family member or nominated representative and there is evidence that this practice is acceptable [38–41]. Return of results to family is also congruent with the importance participants in this study placed on information for family. Thus, an intergenerational approach to SF return, recognising potential benefits to relatives, could present opportunities to better understand penetrance and potential protective genetic factors, as well as impact health outcomes for relatives [5, 6].

### Limitations

Not all people responded to the offer of genetic results, and some did not have their results diagnostically confirmed, so the findings do not represent the views of these individuals. People with different views may also not have responded to the invitation to interview. Participants in this study all spoke English, and the majority received results relating to hereditary breast/ovarian cancer. ASPREE participants are also particularly engaged and reported very positive attitudes towards the research programme. These factors may limit the transferability of findings to other groups and settings. While appropriate to answer the research question, these findings represent the views of research participants, who are only one stakeholder in the debate around offering SFs for the benefit of family members. Further research to understand the views and impact of this practice on family members is recommended.

## Conclusion

Research participants in this qualitative study valued receiving unexpected SFs from the ASPREE trial, particularly because of the potential benefits for family members. These older individuals reported their age and life stage as beneficial in the context of receiving SFs and reported that the provision of SFs enhanced their research participation. The findings of this study suggest that many older research participants would be motivated to receive genetic results, challenging assumptions about the diminishing value of genetic information in this older age group. Given the potential familial and personal benefits of receiving genetic information, decisions to exclude older individuals based on their age must be carefully considered.

## Supplementary information

Interview guide

## Data Availability

Given the small sample size and detailed nature of the qualitative data, the data are not publicly available. The authors will make relevant, unpublished short segments of interviews available for the purposes of verifying or contextualising conclusions on request.
