# Supplementary material for: Older research participants are motivated to receive genetic results for the benefit of younger relatives
Source: Eur J Hum Genet. 2025 Sep 22;34(4):467–73. doi: 10.1038/s41431-025-01940-8 (PMC13046791; doi:10.1038/s41431-025-01940-8)
Supplement: Supplementary file 1 — Interview guide [file 41431_2025_1940_MOESM1_ESM.pdf]

Interview guide for ASPREE psychosocial study

Preamble: This interview is confidential and will be recorded with your permission. You can stop at any time and skip any questions you do not want to answer.
Your information will be stored confidentially and securely and the results of this study will not be shared or published in a way that might identify you.
Please state your full name and date of birth. Do you consent to participating in this research project?

**Themes**	**Relevant to**	**Main question**	**Probe/follow-up**
ASPREE participation	Participant<br>NOK	Could you tell me about your participation in ASPREE?<br>What (if anything), do you know about your relative's participation in ASPREE?	When, how, why…
Expectations of research team<br>Consent to **ASPREE**	Participant	When you consented to ASPREE, did you expect them to find anything?<br>What did you expect of the research team if they found something?<br>What do you think of the consent process now after receiving your results?	Was it adequate? How would you improve it?
Motivation	Participant/NOK	What factors influenced your decision to hear about your results	
Experience of receiving research results<br>Experience of TGC	Participant/NOK	What was it like to get your results from **ASPREE**?<br>How do you think about your results now?<br>How did you find the notification letter and TGC service?<br>What would it be like if you didn't have TGC?	(Un)expected? How?<br>Change over time? How do you think about the value of the information?<br>Vague? Adequate? How did the letter influence your next steps?
Risk perception/management	Participant<br><br>NOK<br><br>Participant/NOK	Since getting your results from ASPREE, how have you thought about your cancer risk?<br>Since getting your notification letter from ASPREE, have you pursued genetic testing for yourself/have your family members pursued genetic testing?<br>What kinds of risk management have you considered/engaged in?	Does this worry you? How often do you think about it?
Family communication	Participant/NOK	Have you talked about results from **ASPREE** with your family? What was it like telling them?	Who have you told? How did they respond? What have they done now?
